# Investigation of Graphene Single Layer on P-Type and N-Type Silicon Heterojunction Photodetectors

**DOI:** 10.3390/s24186068

**Published:** 2024-09-19

**Authors:** Carmela Bonavolontà, Antonio Vettoliere, Marianna Pannico, Teresa Crisci, Berardo Ruggiero, Paolo Silvestrini, Massimo Valentino

**Affiliations:** 1 CNR-ISASI, Institute of Applied Sciences and Intelligent Systems, Via Campi Flegrei 34, I-80078 Pozzuoli, Italy; antonio.vettoliere@isasi.cnr.it (A.V.); massimo.valentino@isasi.cnr.it (M.V.); 2 CNR-IPCB, Institute of Polymers, Composites and Biomaterials, Via Campi Flegrei 34, I-80078 Pozzuoli, Italy; marianna.pannico@cnr.it; 3 CNR-ISASI, Institute of Applied Sciences and Intelligent Systems, Via Pietro Castellino 111, I-80131 Napoli, Italy; 4 DMF—Department of Mathematics and Physics, Università della Campania “L. Vanvitelli”, I-81100 Caserta, Italy

**Keywords:** photodetectors, heterojunction, graphene

## Abstract

Photodetectors are of great interest in several technological applications thanks to their capability to convert an optical signal into an electrical one through light–matter interactions. In particular, broadband photodetectors based on graphene/silicon heterojunctions could be useful in multiple applications due to their compelling performances. Here, we present a 2D photodiode heterojunction based on a graphene single layer deposited on p-type and n-type Silicon substrates. We report on the electro-optical properties of the device that have been measured in dark and light conditions in a spectral range from 400 nm to 800 nm. The comparison of the device’s performance in terms of responsivity and rectification ratio is presented. Raman spectroscopy provides information on the graphene single layer’s quality and oxidation. The results showcase the importance of the doping of the silicon substrate to realize an efficient heterojunction that improves the photoresponse, reducing the dark current.

## 1. Introduction

The increasing interest in the development and strengthening of the performance of light detectors has led to exploring new materials which could replace traditional ones in silicon-based photodetectors (PDs). In recent years, one of the most promising 2D materials has been represented by graphene (Gr), which has attracted enormous attention owing to its electrical, mechanical, and thermal properties [1], which makes it promising for a variety of applications, such as solar-cells, field effect transistors, and photodetectors [2].

Generally, traditional optoelectronic devices based on commercial Si have already become an important core in the integrated circuit industry [3]. Silicon has an indirect energy bandgap of 1.12 eV, and is usually promised to detect light from the visible range to the near-infrared (IR) wavelength (0.4–1.1 μm). However, the photoresponse range is greatly restricted due to the recombination of electron–hole photogenerated pairs [4]. Therefore, it is necessary to develop some alternative materials to improve the performance of photodetectors, including new optoelectronic materials.

Graphene, due to its unique two-dimensional structure (2D) and versatile electronic and optical properties, has attracted significant attention in the development of high-performance optoelectronic devices [5,6,7,8,9,10,11,12,13].

Due to the low optical absorption and high conductivity of graphene, the low photocarriers generated in the graphene have a lifetime of few picoseconds. Therefore, it is necessary to separate the electron–hole pairs within a time scale less than the carrier’s lifetime to generate a considerable photocurrent. Consequently, graphene is not used as an absorption medium, but as a transparent electrode to collect the photoexcited carriers generated into the semiconductor substrate. A promising platform for photodetectors and sensors has been provided: transferring graphene onto a semiconductor such as silicon [5,14,15,16,17]. Integrating graphene with silicon to form heterojunctions is one of the strategies to improve the lifetime of the photogenerated carriers. In a graphene/Si-based photodetector, silicon absorbs photons into the depletion region, generating electron–hole pairs that will drive by the build-in electric field within graphene into external circuit. Moreover, to reduce the leakage current of the graphene/Si interface, the insertion of an oxide layer has been recognized to be useful to improve the photodetectors’ performance [18,19]. Recently, photodetectors based on graphene/oxide/Si heterojunctions have been developed, which exhibit high responsivity and detectivity [18].

More specifically, heterostructures involving 2D materials, such as the graphene single layer, allow heterointerfaces that play a pivotal role in altering the optical properties of dissimilar materials to be achieved [20,21,22], facilitating the electronic band alignment and the optical transactions between different materials, as well as improving the effective spatial separation of electron–hole pairs and tunneling efficiency [23,24]. The conduction mechanism involved in the graphene/Si heterojunctions depends significantly on the graphene Fermi level position with respect to the Dirac point. The exposure of graphene to air and humidity induces an oxidation process that corresponds to the p-type doping of the graphene [25]. The excess of holes in the graphene lowers its Fermi level below the Dirac point, enhancing the injection of carriers across the heterojunction, which reduces the accumulation of charge and the depletion region across the heterojunction [26,27,28]. 

In this work, experimental results concerning the performance of heterojunctions based on n-type and p-type silicon substrates covered by graphene monolayer (SLGr) are reported. The electro-optical properties of the realized photodetectors are presented. The oxidation process, due to air and ambient conditions, that led to a p-type doping of the graphene monolayer was revealed by the Raman spectroscopy analysis. The experimental results demonstrate the potential of the graphene monolayer to improve the detection capability of PDs based on Si heterojunctions.

## 2. Materials and Methods

P-type and n-type silicon wafers, with thicknesses of 300 µm, were covered by a 60 nm thick silicon nitride (Si_3_N_4_) layer deposited by plasma-enhanced chemical-vapor deposition (PECVD). On the back side of the device for the p-type and n-type silicon wafers, thin layers of Si p+ and Si n+ were deposited, respectively. The back electrode was represented by a Ti (20 nm)/Pt (100 nm) layer, which, in contact with the p+ and n+ layers, defined an ohmic junction on the back side of the structures. On the top of Si_3_N_4_, two circular Pt/Ti electrodes with diameters of 1 mm at 4 mm away each other were deposited.

**Figure 1:**
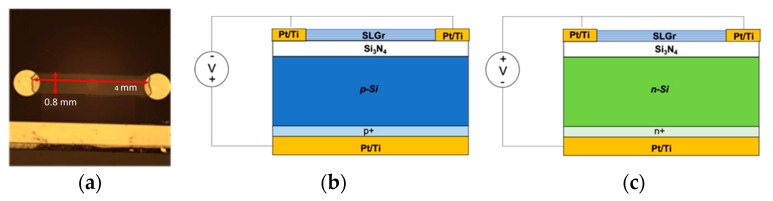
(**a**) Image of the SLGr present on the top surface of the devices (red lines report the width and length of the SLGr transferred on the substrate); (**b**,**c**) illustration of the devices’ structures (SLGr/p-Si heterostructure and SLGr/n-Si heterostructure, respectively). On the back side of the structure, both the devices have thin layers of Si p+ and Si n+ to guarantee ohmic contact between the silicon wafer and the Pt/Ti electrodes.

The top surface of the devices exhibited a single layer of graphene (SLGr) transferred on the Si_3_N_4_ layer between the Pt/Ti electrodes, covering an active area of 3.2 mm^2^, as reported in Figure 1a. The SLGr with a thickness of about 1 nm, covered with a 500 nm thick poly (methyl methacrylate) (PMMA) film and sustained by a polymer substrate, was purchased from ACS Material (Pasadena, CA, USA) and transferred onto Si-based substrates using the following procedure. First, the substrate was dipped in deionized (DI) water, where the PMMA–graphene film was released, floating on the water surface. Then, the floating PMMA–graphene film was picked up from the water using the silicon-based substrate. After the transfer, the graphene-based substrate was dehydrated, blown dry using a nitrogen gun for a few minutes, and then heated on a hot plate. Finally, the sample was steeped in acetone to dissolve the PMMA and patterned by O_2_ plasma etching after a photolithographic process to produce the desired layout [29].

The Raman spectra were collected by a confocal Raman micro spectrometer (XploRA-Plus, Horiba-Scientific, Kyoto, Japan) operating with a diode laser excitation source of 532 nm corresponding to 80 mW. The 180° back-scattered radiation was collected by an Olympus metallurgical objective (MPlan 100×, NA = 0.90) with confocal and slit apertures set to 300 and 100 μm, respectively. A grating with 1200 grooves/mm was used throughout to disperse the scattered light with the highest spectral resolution. The radiation was focused onto a CCD detector (Horiba Cameras, Mod. Syncerity, Kyoto, Japan) cooled at −65 °C by a Peltier module. The spectra, calibrated using a monocrystalline silicon wafer as a reference (primary Raman mode at 520 cm^−1^), were collected with an exposure time of 1 s in the Raman-shift range of 1100 ÷ 3200 cm^−1^. Raman imaging measurements were performed in the mapping mode: the sample was placed on a piezo-electrically driven microscope-stage with a x, y resolution of 10 ± 0.5 nm and a z resolution of 15 ± 1 nm. The stage was scanned at a constant speed in the x–y plane over a 200 × 200 μm^2^ area with a 20 μm step size.

Figure 1b,c show the devices stacking sequence and the voltage bias applied to obtain the current–voltage (I-V) characteristics. This configuration allowed us to reveal the current that drifts across the heterojunction.

The photoresponse measurements were carried out using an Oriel 77501 Fiber Optic Illuminator with a 100-Watt Quartz Halogen Lamp at a wavelength ranging between 400 nm and 700 nm with the power tuned from 1 to 3.5 µW. The I-V characteristics were measured using a voltage supply (Source Meter Keithley, mod. 2635) and a picoammeter (Keithley Dual-channel, mod. 6482).

## 3. Results and Discussion

**Figure 2:**
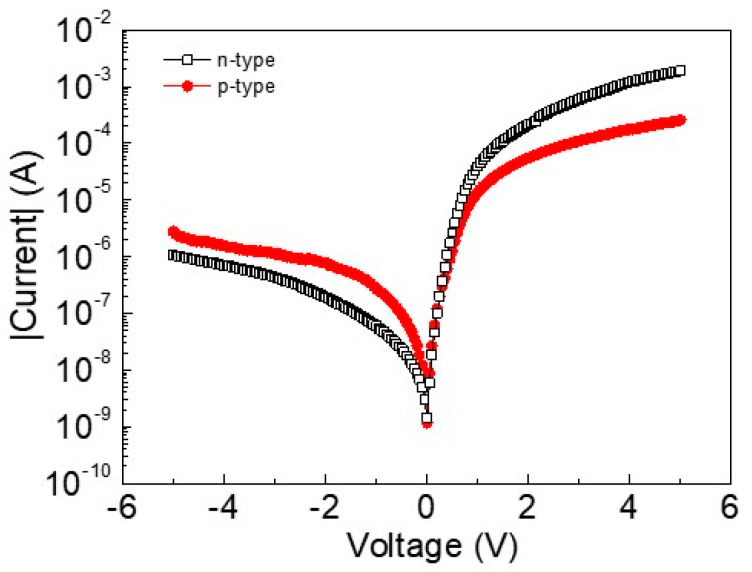
I-V curve of SLGr-based devices in dark conditions in the voltage range between −5 V and 5 V.

The I-V curve in dark conditions was measured between −5 V and 5 V, as reported in Figure 2. The graph shows some similarities and differences regarding the current intensity. In the forward polarization (positive voltage), the current of SLGr/p-type Si was lower than that of the SLGr/n-type Si device, while in the reverse configuration (negative voltage), an opposite situation appeared: the SLGr/n-type Si had the lowest dark current. Both devices exhibited clear rectifying behavior and dark currents lower in the reverse than in the forward configuration. The photo-to-dark current ratios were estimated to be 10^2^ and 10^3^ for the SLGr/p-type and n-type devices, respectively.

It is generally claimed that, in the forward polarization, the accumulation stage is experienced: Near the Si_3_N_4_/Si interface, an accumulation of the majority carriers arises [27]. However, the two Si substrates are characterized by different doping (n-type and p-type), which imply different work functions that play important roles in explaining the measured dark currents in the two devices.

**Figure 3:**
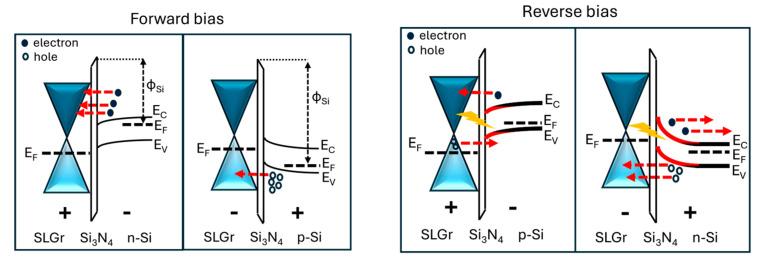
Energy band diagrams of the graphene/ Si_3_N_4_/Si heterojunctions in forward and reverse bias configuration.

Then, considering that the n-type Si has a lower work function than that of the p-type Si substrate, it is established that, across the junction SLGr/Si_3_N_4_/n-Si, there is a flow of electrons from the n-Si to the SLGr, resulting in a smaller accumulation current at the Si_3_N_4_/n-Si interface and a significant dark current in the device. On the other hand, the larger work function of the p-type with respect to the n-type silicon substrate reduces the flow of holes from Si to SLGr, resulting in a significant accumulation charge at the Si_3_N_4_/p-Si interface and a reduced dark current flow [27]. These conduction mechanisms are sketched in Figure 3 in the box related to the forward bias configuration.

In the reverse configuration, as shown in Figure 2 (negative voltage), the dark currents of the p-type and n-type Si substrate experience an opposite trend. The n-type-based device has a lower dark current with respect to the other device. This finding could be ascribed to the presence of the depletion region induced by the reverse bias voltage. The different current intensity suggests that the depletion regions in the two devices have different widths. 

Generally, the depletion width across a heterojunction depends on the accumulation of the minority charge density at the Si_3_N_4_/Si interface in correspondence with the conduction and valence band of the p-type and n-type Si-based devices [27,30]. With this aim, the insertion of the Si_3_N_4_ layer increased the interfacial barrier, thus suppressing the dark current, thereby improving the accumulation across the heterojunction interface. The greater the accumulation of charge, the greater the bending upward or downward of the bands near the interface. Moreover, the charges that drift to the interface depend on the potential barrier due to the position of the Fermi level in the graphene and the silicon substrate. Consequently, the narrow depletion width in the p-type based device could be due to a lower graphene Fermi level that produces an excess of holes that quickly recombine with the electrons, reducing their accumulation near the p-type Si interface. This mechanism is reported in Figure 3 in the sketch related to the reverse bias configuration, where the different depletion region widths that characterize the two devices are reported. In the case of p-type silicon, there is a flow of holes from the graphene single layer to the silicon substrate, leading to easy recombination between the holes and the photogenerated electrons. On the other hand, in the case of the n-Si substrate, the larger depletion region and the position of the graphene Fermi level with respect to the silicon reduce the recombination, and under illumination, the build-in potential due to the depletion region width drifts the photogenerated charges efficiently, suppressing their recombination. 

**Figure 4:**
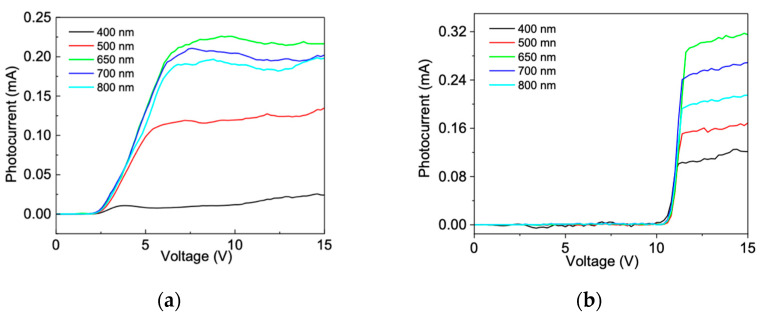
I-V characteristic of (**a**) SLGr/p-type and (**b**) SLGr/n-type devices under illumination from 400 nm to 800 nm with a power of 0.5 mW.

This assumption is supported by the photocurrent measurements reported in Figure 4, where the photocurrents versus the reverse bias of the two devices under illumination from 400 nm to 800 nm powered at 0.5 mW are depicted. One of the common features of the photocurrent curves is the voltage threshold at about 2 V and 12 V for the SLGr/p-type and SLGr/n-type heterojunctions, respectively. It could be noted that, in both devices, these thresholds do not depend on the light source wavelength.

A more detailed look at Figure 4a reveals that the photocurrent of the SLGr/p-type device shows a steady low value until about 2 V. From this point, on it increases rapidly until a rather steady trend characterized by fluctuations is reached. Clearly, there are similar trends for the photocurrent of the SLGr/n-type device reported in Figure 4b: It stayed at low value until about 12 V; then, it rose sharply to reach a gradual upward trend.

It could be noted that one of the key features of the photocurrent is the voltage threshold, which represents the bias voltage necessary for the charges to overtake the depletion field across the heterojunction. The findings suggest that, in the SLGr/n-type device, it is necessary to apply a large external bias (12 V) to allow for the charge transfer across the heterojunction from the silicon to the SLGr layer. On the contrary, in the p-type-based device, it is sufficient to apply a lower voltage bias, about 2 V, to inject the charge into the SLGr layer. According to the data, it is reasonable that, in the n-type- and p-type-based devices, different depletion widths are present. To substantiate this issue, an estimation of the depletion width was obtained considering the silicon substrate resistivity [27]. The depletion width was calculated to be 2 μm at 2 V and 80 μm at 10 V for the SLGr/p-type and SLGr/n-type devices, respectively. This result demonstrates that the n-doped silicon substrates produce larger depletion widths with respect to the p-type Si substrate. 

**Figure 5:**
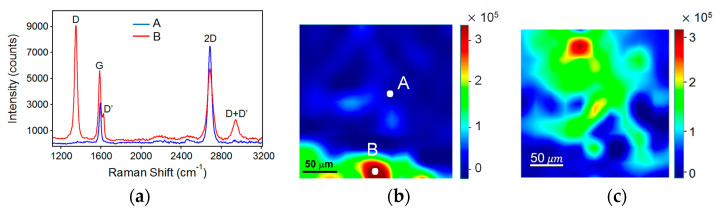
(**a**) Raman image related to the D-band peak at 1345 cm^−1^ collected on an area of 200 × 200 µm^2^; (**b**) the Raman spectra collected at points A and B; (**c**) Raman image related to the D-band peak at 2684 cm^−1^ collected in an area of 200 × 200 µm^2^.

On the whole, a drop in the graphene Fermi level could occur with a possible p-type doping of the graphene caused by the air and humidity molecules. In our case, the graphene single layer was directly exposed to the environment, so it was reasonable to consider a p-doped effect, which could lead to a p-n and p-p junction on n-Si and p-Si substrates, respectively [25,31,32]. This feature is evidenced by the Raman spectroscopy reported in Figure 5.

The Raman spectroscopy was carried out in mapping mode on several regions of the SLGr/n-type. Figure 5a shows the Raman spectra (corrected for the baseline) related to the two points, A and B, depicted on the map, which were collected over an area of 200 × 200 µm^2^, as reported in Figure 5b.

The spectrum collected at point A shows the most prominent peaks characteristic of graphene: The G band at 1586 cm^−1^, corresponding to the E_2g_ phonon at the Brillouin zone center, which is characteristic of sp^2^-bonded carbon atoms in a two-dimensional hexagonal lattice, and the strong 2D band at 2690 cm^−1^, resulting from a second-order two-phonon process [33]. Their intensity ratio I_2D_/I_G_ was calculated to be ~2, indicating that the transferred graphene is a single layer [34,35]. 

In the spectrum collected at point B, new peaks appeared at 1345 cm^−1^ (D band) and at 1620 cm^−1^ (D′ band). The D peak is associated with the breathing modes of sp^2^ carbon rings, and the D′ peak represents the in-plane longitudinal optical (LO) phonons at the K point [36]. The presence of both D and D′ peaks is evidence for the existence of defects in the graphite layer, such as bond angle disorder, bond length disorder, vacancies, edge defects, etc. [37]. Their combination peak (D + D′), observed around 2940 cm^−1^, is a result of the overtone of the D and D′ bands, further confirming the presence of edge defects and disorder in the graphene structure [36].

Furthermore, the disorder and defect distribution could be evidenced by mapping the peak intensity of D-band at 1345 cm^−1^, as reported by the Raman image in Figure 5b. The map highlights a non-uniform distribution of defects in localized areas of the graphene single layer’s surface. These defects represent some wrinkles on the graphene surface; there is no absence of graphene, so they do not modify the electrical conduction properties of the layer. 

Another important feature in the Raman spectra is the presence of a 2D band at 2684 cm^−1^ (also called the G’ band); this is the overtone of the D band [38]. Generally, the FWHM (Full Width at Half Maximum) of the 2D band broadens as a function of defects: The spectrum in Figure 5a demonstrates that the 2D band broadens to about 60 cm^−1^ (point B) compared to 40 cm^−1^ (point A). In most cases, the 2D band is used to evaluate the structural parameters of the c-axis orientation, since this band is very sensitive to the stacking order of the graphene along the c-axis [39]. It is expected to be a reduction in the 2D-band intensity due to the breaking of the stacking order by the oxidation reaction [40]. 

In Figure 5c, the 200 × 200 µm^2^ map of the 2D-band intensity is depicted. The image demonstrates that the graphene monolayer presents clearly oxidized regions in which the 2D-band intensity is reduced. Then, according to the literature [34,40,41], the significant reduction in intensity and broadening of the 2D band related to the G-band and 2D-band are associated with the hole-doping process by oxygen absorption, which confirms the p-type doping of the graphene monolayer. 

All the findings evidenced by Raman spectroscopy clearly provide information on the oxidation process, which does not correspond to a poor quality of the graphene layer, but rather implies the p-type doping of the graphene single layer. Consequently, it is reasonable to consider the heterojunctions SLGr/p-Si and SLGr/n-Si as p-p and p-n junctions, respectively. Then, the excess of holes in the SLGr affects its Fermi level (shifting it away from the Dirac point), resulting in different depletion region widths in the two devices. In the photoresponse, some features of this result are present, such as the different voltage thresholds and the switching rate of the photocurrent.

As reported in Figure 4, both devices in the range between 400 nm and 800 nm show photoresponses characterized by marked upward trends that reach saturation values with different rates. 

**Figure 6:**
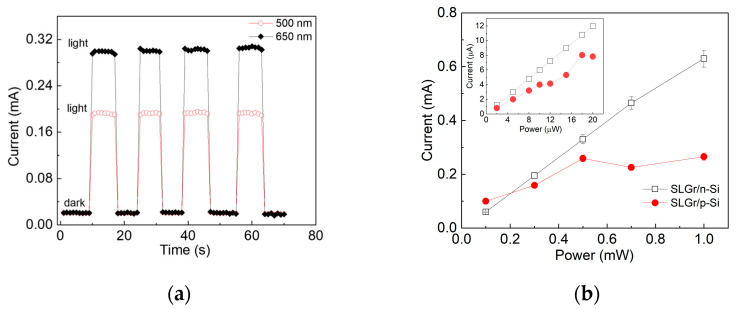
(**a**) Current response of the SLGr/n-Si device to the periodic chopping of light source: 500 nm and 650 nm with a power of 0.5 mW, biasing the devices at 15 V. (**b**) Current as a function of power for the two devices at wavelength of 650 nm in the ranges from 0.1 mW to 1 mW and from 1 μW to 20 μW in the inset.

The I-V curves depicted in Figure 4a show low slew rates with respect to the photocurrent curves in Figure 4b. This finding demonstrates that the photoresponse in the SLGR/n-type device is steady and faster than that of the p-type Si-based device. The latter shows considerable variation in the photocurrent after the upward trend, while the heterojunction based on n-type Si shows a steady saturation photocurrent, which increases as a function of the bias voltage with the same slope for all the wavelengths. To validate the photoresponse reproducibility of the SLGR/n-type, the photocurrent was measured by chopping the light source on and off, as reported in Figure 6.

The current was measured by biasing the device at 15 V and illuminating with 500 nm and 650 nm light sources powered at 0.5 mW. It could be noted that the switching experienced a rapid response (about 4 μs) and a significant reproducibility of the current even after several cycles using different light wavelengths. 

Moreover, measurements of the photoresponse as a function of light power were carried out. An estimation of the linear dynamic range (LDR), defined as the range of incident optical power for which the photodetectors can respond linearly [42], was obtained, with the power ranging from 0.5 μW to 1 mW. The SLGr/n-Si exhibited an LDR about 66 dB larger than that of SLGr/p-Si, which was equal to 26 dB. According to the data reported in Figure 6b, the photocurrent of the SLGr/n-Si device preserved linearity from a few μW to 1 mW, while the presence of the p-type silicon substrate restricted the linearity to only the range of 2–10 μW (see inset of Figure 6b). This result demonstrates that the SLGr/n-Si device provides a better performance in terms of linearity over a broad range of light power.

**Figure 7:**
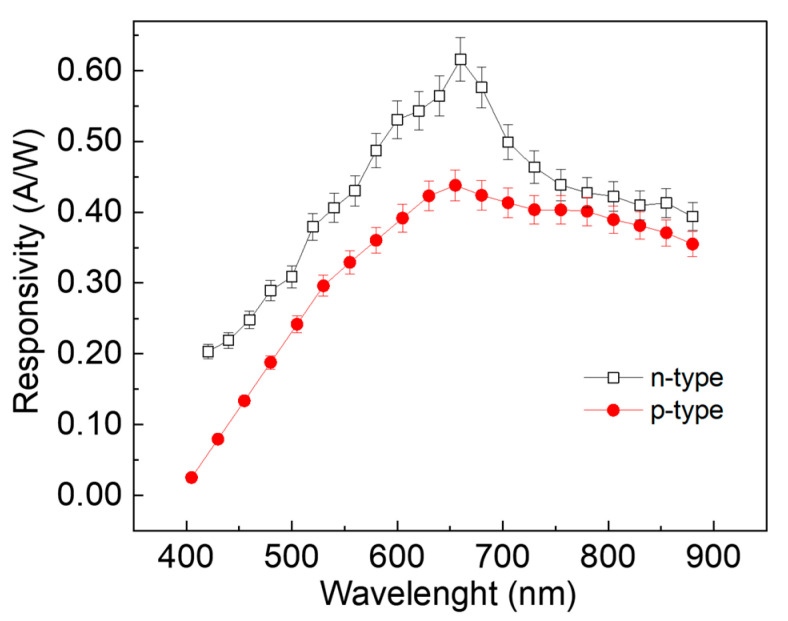
Comparison of the responsivity versus wavelength for both devices.

The photoresponses of the two devices allow us to estimate the responsivity, which represents one of the most important figures of merit for photodetectors. It is defined as the photocurrent-to-optical-power ratio and represents the capability of the device to convert the photons into a current in a specific wavelength range. Figure 7 reports the comparison of the responsivity of the n-type- and p-type-based devices as a function of the wavelength in the range of 400 nm to 800 nm. It could be noted that, even if the two devices had a peak of responsivity at about 650 nm, the SLGr/n-Si device outranged the responsivity of the SLGr/p-Si device, especially in the UV range.

The peak response at about 650 nm for both devices could be related to the silicon substrate, since the photogeneration of charge by the incident light occurred mainly in the silicon. Then, at about 650 nm, the penetration depth of the light radiation was higher than the UV radiation, so that in the visible range, a significant amount of photogenerated charges were present, leading to a higher photocurrent [23]. Additionally, an estimation of the detectivity (D*) and noise equivalent power (NEP) was carried out. The D* describes the capability of the device to detect low light, and it is defined as $D*=R/\surd (2\cdot e\cdot J_{d}),$ where *R*, *e*, and *J_d_* are the responsivity at 650 nm, elementary charge, and dark current density, respectively [18]. Moreover, the NEP is related to the minimum measurable power detected by the device, and it is defined as NEP=√A/D*, where A is the area of the device [22]. The D* and NEP were extracted to be 6.5 × 10^10^ Jones and 8.7 pW/Hz^0.5^ for the SLGr/n-Si, and 4.3 × 10^10^ Jones and 13.2 pW/Hz^0.5^ for the SLGr/p-Si device. 

The responsivity estimation demonstrates that the n-silicon substrate allows for a more significant photodetection efficiency than the p-type based device thanks to its higher capability to reduce the recombination rate of the photogenerated charges. Generally, a low intensity of the dark current and a large depletion region are fundamental in order to obtain a significant photoresponse: the lower the dark current, the better the photocurrent, and the larger the depletion region, the more efficient the photoinduced hole–electron separation. All these features are present in the n-type-based device. Consequently, this device could be considered as a suitable candidate with which to achieve outstanding photodetectors.

## 4. Conclusions

Photodetectors based on Gr/Si junction were realized. A graphene single layer was transferred onto n-type and p-type silicon substrates with a Si_3_N_4_ interfacial layer. The photoresponse capability of the two devices under illumination condition using light sources ranging from 400 nm to 800 nm was investigated. Different voltage thresholds characterized the I-V curves of the two devices. The Raman spectroscopy results suggest that p-doping of the graphene single layer due to air exposure affects the properties of the heterojunction SLGr/Si_3_N_4_/Si in terms of the depletion region width. The SLGr/n-Si device demonstrated a better linear performance, with an LDR of 66 dB with respect to the 26 dB of the SLGr/p-Si device. Furthermore, the responsivity was extracted to be 0.6 A/W at 650 nm and 0.4 A/W at 400 nm for the SLGr/n-Si device. This proves that the responsivity in the UV range of a single layer of graphene on the Si n-type device was widely improved compared with the conventional Si-based photodetectors and the previous device, which was realized by the authors based on a reduced graphene oxide (rGO) layer [23]. 

Finally, the graphene monolayer on the n-type Si-based substrate offered a good photoresponse performance from the UV to the visible wavelength range. Further research is in progress to investigate the performance of devices with large areas covered by graphene single layers, and to examine the properties of the SLGr/Si_3_N_4_/n-Si heterojunction to determine a useful band diagram that supports the photoresponse results and provides deeper knowledge of graphene-based electronics. 

## Data Availability

The data presented in this study are available on request from the corresponding author because the data are part of an ongoing study.
